# Supplementary figures and images for: Developing a core outcome set for periodontal trials
Source: PLoS One. 2021 Jul 22;16(7):e0254123. doi: 10.1371/journal.pone.0254123 (PMC8297801; doi:10.1371/journal.pone.0254123)

**S1 Appendix: Participant information sheet**


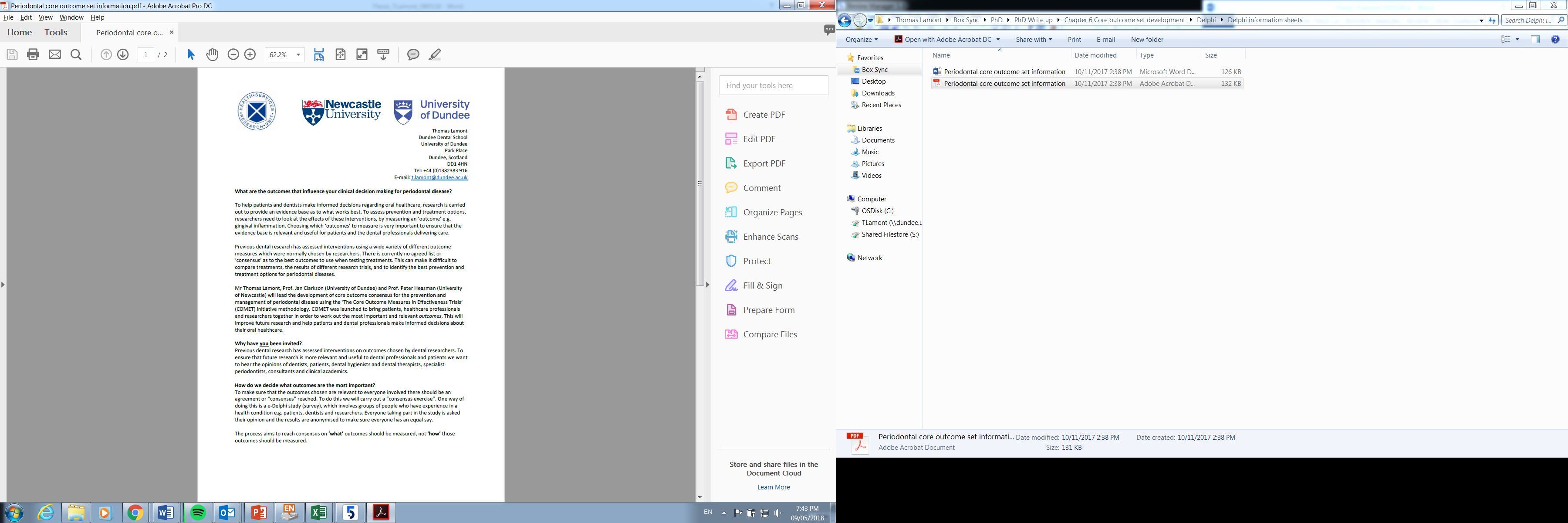


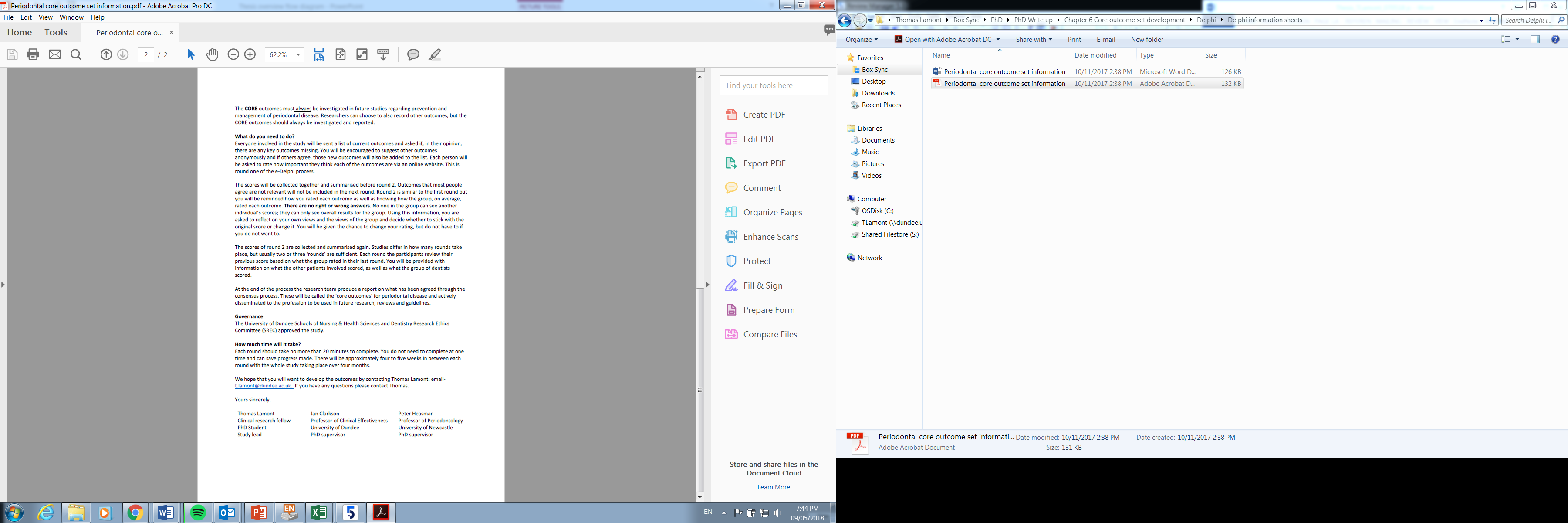

Supplement: S1 Appendix — (DOCX) [file pone.0254123.s001.docx]
