# Supplementary material for: Developing a core outcome set for periodontal trials
Source: PLoS One. 2021 Jul 22;16(7):e0254123. doi: 10.1371/journal.pone.0254123 (PMC8297801; doi:10.1371/journal.pone.0254123)
Supplement: S1 Table — (DOCX) [file pone.0254123.s002.docx]

**S1 Table. Included Cochrane reviews and protocols**

| Cochrane review/protocol | Number of studies | Number of participants  in review |
| --- | --- | --- |
| 1. Powered versus manual toothbrushing for oral health (review) | 56 | 4624 |
| 1. Different powered toothbrushes for plaque control and gingival health (review) | 17 | 1369 |
| 1. Flossing for the management of periodontal diseases and dental caries in adults (review) | 12 | 1083 |
| 1. Triclosan/copolymer containing toothpastes for oral health (Review) | 30 | 14835 |
| 1. Interdental brushing for the prevention and control of periodontal diseases and dental caries in adults (review) | 7 | 354 |
| 1. Chlorhexidine mouthrinse as an adjunctive treatment for gingival health (protocol) | Protocol | |
| 1. Full-mouth treatment modalities (within 24 hours) for chronic periodontitis in adults (review) | 12 | 389 |
| 1. Routine scale and polish for periodontal health in adults (review) | 3 | 836 |
| 1. One-to-one oral hygiene advice provided in a dental setting for oral health (protocol) | Protocol | |
| 1. Oral health educational interventions for nursing home staff and residents (protocol) | Protocol | |
| 1. Psychological interventions to improve adherence to oral hygiene instructions in adults with periodontal diseases (Review) | 4 | 344 |
