# Supplementary material for: Developing a core outcome set for periodontal trials
Source: PLoS One. 2021 Jul 22;16(7):e0254123. doi: 10.1371/journal.pone.0254123 (PMC8297801; doi:10.1371/journal.pone.0254123)
Supplement: S2 Table — Pre-specified primary and secondary outcomes of included Cochrane reviews and protocols. (DOCX) [file pone.0254123.s003.docx]

**S2 Table. Outcomes identified in Cochrane reviews and protocols (July 2016)**

| Cochrane review/protocol | Primary outcome | Secondary outcome |
| --- | --- | --- |
| 1. Powered versus manual toothbrushing for oral health (review) | 1. Quantified levels of plaque 2. Quantified levels of gingivitis | 1. Calculus 2. Staining 3. Dependability and cost of the brush used, including mechanical deterioration 4. Adverse effects such as hard or soft tissue injury and damage to orthodontic appliances and prostheses |
| 1. Different powered toothbrushes for plaque control and gingival health (review) | 1. Quantified levels of plaque 2. Quantified levels of gingivitis | 1. Cost 2. Reliability 3. Calculus 4. Staining 5. Adverse events |
| 1. Flossing for the management of periodontal diseases and dental caries in adults (review) | 1. Periodontal disease, assessed by gingivitis indices (both inflammatory and bleeding) 2. Interproximal caries, assessed by (a) progression of caries into enamel or dentine, and (b) change in decayed, missing and filled tooth surfaces (D(M)FS) index 3. Harms and adverse effects 4. Plaque indices 5. Calculus indices 6. Clinical attachment loss 7. Quality of life | 1. Economic and resource cost of flossing 2. Bad breath (halitosis) |
| 1. Triclosan/copolymer containing toothpastes for oral health (Review) | 1. Plaque levels measured using any appropriate scale 2. Gingival health measured using any appropriate scale | 1. Incidence of periodontitis 2. Caries: a) new incidence, and b) caries increment - change in decayed, missing and filled surfaces (DMFS/dmfs) index. 3. Calculus measured using any appropriate scale 4. Adverse effects (e.g. taste disturbance, staining, allergic reaction, etc.) 5. Participant-centred outcomes: a) participant-assessed quality of life scores, and b) participant satisfaction with product |
| 1. Interdental brushing for the prevention and control of periodontal diseases and dental caries in adults (review) | 1. Gingivitis - assessed by gingival indices (both inflammatory and bleeding) 2. Periodontitis - assessed by clinical attachment loss 3. Interproximal caries - assessed by (a) progression of caries into enamel or dentine, (b) change in decayed, missing and filled tooth surfaces (D(M)FS) index, (c) radiographic evidence 4. Plaque indices. 5. Harms and adverse effects. | 1. Bad breath (halitosis) 2. Quality of life |
| 1. Chlorhexidine mouthrinse as an adjunctive treatment for gingival health (protocol) | 1. Gingivitis measured using an appropriate index | 1. Dental plaque measured using an appropriate index 2. Adverse effects including but not limited to: 3. Tooth staining, 4. Supragingival calculus formation 5. Changes in taste perception 6. Parotid gland swelling 7. Irritation of the oral mucosa |
| 1. Full-mouth treatment modalities (within 24 hours) for chronic periodontitis in adults (review) | 1. Tooth loss 2. Change in probing pocket depth after three to four months and six to eight months | 1. Change in clinical attachment level after three to four months and six to eight months 2. Change in bleeding on probing after three to four months and six to eight months 3. Adverse events |
| 1. Routine scale and polish for periodontal health in adults (review) | 1. Periodontal disease, assessed by gingivitis indices (both inflammatory and bleeding). | Clinical status factors   1. Calculus and plaque indices. 2. Changes in probing depth. 3. Changes in attachment level. 4. Periodontal indices. 5. Tooth loss. 6. Adverse events.   Patient-centred factors   1. Halitosis. 2. Patient satisfaction with oral comfort. 3. Patient satisfaction with appearance (including gingival recession). 4. Patient satisfaction with actual care received. 5. Patient satisfaction with provider of care (i.e. dentist, therapist or hygienist).   Economic cost factors   1. Economic and resource cost of scale and polish. |
| 1. One-to-one oral hygiene advice provided in a dental setting for oral health (protocol) | 1. Oral cleanliness (e.g. plaque levels) 2. Periodontal health (e.g. presence of gingivitis and probing depths) 3. Caries (e.g. dmft/DMFT or other indices) 4. Oral infection (e.g. candidiasis, acute necrotising ulcerative gingivitis). | Patient-centred factors   1. Patient-reported behaviour changes (e.g. tooth brushing/flossing/mouthwash use) 2. Patient-reported health indices 3. Patient satisfaction with provider of advice (e.g. dentist, therapist, hygienist, dental nurse) 4. Patient satisfaction with advice format 5. Patient-reported changes in knowledge, attitudes and quality of life following provision of advice.   Economic factors   1. Cost effectiveness 2. Other outcomes |
| 1. Oral health educational interventions for nursing home staff and residents (protocol) | 1. Oral health-related quality of life 2. Oral health 3. Caries, incidence of new caries 4. Dental or denture plaque or both 5. Gingivitis | 1. Nutritional status (e.g. body weight, Body Mass Index - BMI). 2. Incidence of respiratory diseases and pneumonia. 3. Adverse effects of the interventions. 4. Oral health-related knowledge of staff or residents or both 5. Oral health-related attitude and behaviour of staff or residents or both |
| 11. Psychological interventions to improve adherence to oral hygiene instructions in adults with periodontal diseases (Review) | 1. Bleeding on probing or gingivitis score 2. Any other clinical markers of periodontal diseases (for example, plaque score, probing depths, clinical attachment loss, recession, BPE (basic periodontal examination) scores | 1. Self-reported measures of oral health-related behaviour 2. Self-reported beliefs about and attitudes to oral health-related behaviour |

Legend: Pre-specified primary and secondary outcomes of included Cochrane reviews and protocols
