# Supplementary material for: Developing a core outcome set for periodontal trials
Source: PLoS One. 2021 Jul 22;16(7):e0254123. doi: 10.1371/journal.pone.0254123 (PMC8297801; doi:10.1371/journal.pone.0254123)
Supplement: S3 Table — Unique de-deduplicated outcomes and the review and protocol numbers that reported these outcomes. Numbers used provided in S2 Table. (DOCX) [file pone.0254123.s004.docx]

**S3 Table. Unique outcomes reported in Cochrane review and included trial**

| Outcome | Outcome included in review  (review number) | Reported by at least one trial included in review  (review number) |
| --- | --- | --- |
| 1. Quantified levels of plaque | 1,2,3,4,5,6,8,9,10,11 | 1,2,3,4,5,7,8,11 |
| 2. Quantified levels of gingivitis | 1,2,3,4,5,6,7,8,9,10,11 | 1,2,3,4,5,7,8,11 |
| 3. Dental Caries | 3,4,5,9,10 | 4 |
| 4. Harms and adverse effects | 1,2,3,4,5,6,7,8,10 | 1,3,4,5,7 |
| 5. Calculus | 1,2,3,4,6,8 | 1,4,8 |
| 1. Changes to perception | 6 | Not reported |
| 7. Clinical attachment loss/Changes in attachment level | 3,5,7,8,11 | 1,4,5,7,8,11 |
| 8. Probing depths | 7,8,9,11 | 1,5,7,8 |
| 9. Staining | 1,2,4,6 | 1,3,4 |
| 10. Oral infection | 9 | Not reported |
| 11. Incidence of periodontitis | 4 | Not reported |
| 12. Bad breath (halitosis) | 3,5,8 | Not reported |
| 13. Tooth loss | 7,8 | Not reported |
| 14. Cost | 1,2,3,8,9 | Not reported |
| 15. Reliability | 2 | Not reported |
| 16. Quality of life | 3,4,5,10 | Not reported |
| 17. Satisfaction with product | 4 | Not reported |
| 18. Satisfaction with appearance | 8 | Not reported |
| 19. Satisfaction with actual care received | 8 | Not reported |
| 20. Satisfaction with provider of care | 8,9 | Not reported |
| 21. Patient reported behaviour change | 9,10,11 | 11 |
| 22. Patient reported health | 9,10 | Not reported |
| 23. Patient reported change in knowledge | 9,10 | 11 |
| 24. Nutritional status | 10 | Not reported |
| 25. Respiratory disease | 10 | Not reported |

Legend: Unique de-deduplicated outcomes and the review and protocol numbers that reported these outcomes . Numbers used provided in S3 Table.
