# Supplementary material for: Developing a core outcome set for periodontal trials
Source: PLoS One. 2021 Jul 22;16(7):e0254123. doi: 10.1371/journal.pone.0254123 (PMC8297801; doi:10.1371/journal.pone.0254123)
Supplement: S4 Table — Additional outcomes reported by trials but not included in Cochrane review. Review and protocol numbers used provided in S2 Table. (DOCX) [file pone.0254123.s005.docx]

**S4 Table. Additional unique outcomes reported in the included trials of the Cochrane reviews.**

| Additional outcomes reported in trials | Included in review number: |
| --- | --- |
| 1. Microbiological parameters | 1,4,7 |
| 1. Recession | 1,5,7 |
| 3. Abrasion | 1,3 |
| 4. Wear of toothbrushes | 1 |
| 5. Intra-crevicular exudate | 3 |
| 6. Dental crown failure | 4 |
| 7. Compliance | 5 |
| 8. Relative interdental papillary level | 5 |
| 9. Average pain scores | 7 |
| 10. Body temperature | 7 |
| 11. Analgesics required | 7 |
| 12. Self-efficacy beliefs | 11 |

Legend: Additional outcomes reported by trials but not included in Cochrane review. Review and protocol numbers used provided in S3 Table.
