# Supplementary material for: Developing a core outcome set for periodontal trials
Source: PLoS One. 2021 Jul 22;16(7):e0254123. doi: 10.1371/journal.pone.0254123 (PMC8297801; doi:10.1371/journal.pone.0254123)
Supplement: S5 Table — (DOCX) [file pone.0254123.s006.docx]

**S5 Table. Additional ‘missing’ outcomes suggested by e-Delphi participants.**

| **Additional Outcomes** | **Domain** |
| --- | --- |
| 1. Bone levels on radiographic examination | Clinical |
| 1. Endodontic status | Clinical |
| 1. Functional occlusion | Patient orientated |
| 1. Furcation Involvement | Clinical |
| 1. Glycaemic control | Clinical |
| 1. Manual dexterity | Patient orientated |
| 1. Measure of obesity | Clinical |
| 1. Patient stress | Patient orientated |
| 1. Smoking status | Clinical |
| 1. Tooth migration | Clinical |
| 1. Tooth mobility | Clinical |
