# Supplementary material for: Developing a core outcome set for periodontal trials
Source: PLoS One. 2021 Jul 22;16(7):e0254123. doi: 10.1371/journal.pone.0254123 (PMC8297801; doi:10.1371/journal.pone.0254123)
Supplement: S6 Table — A measure of the average change in score between each round of the e-Delphi study. (DOCX) [file pone.0254123.s007.docx]

**S6 Table. Stability of opinion scores between e-Delphi round 1 and 2 for each outcome across stakeholder groups.**

| **Outcomes that were included in round 1 and 2** | **Stability of scores** | |
| --- | --- | --- |
|  | **Patients** | **Dental Professionals and Researchers** |
| Abrasion | -0.1 | -0.2 |
| Average pain scores | -0.9 | -0.2 |
| Calculus | -1.9 | 0.2 |
| Clinical attachment loss | -1.7 | 0.3 |
| Compliance | 0.0 | 0.2 |
| Cost | -0.3 | -0.3 |
| Dental caries | -0.3 | -0.1 |
| Dental crown failure | -0.9 | -0.6 |
| Incidence of periodontitis | -0.1 | 0.4 |
| Intra-crevicular exudate | -1.7 | -0.6 |
| Irritation of oral mucosa | -1.8 | -0.3 |
| Microbiological parameters | -1.6 | -0.7 |
| Oral infection | -0.6 | -0.7 |
| Patient reported behaviour change | -1.1 | -0.1 |
| Patient reported change in knowledge | -0.9 | 0.0 |
| Patient reported health | -0.8 | -0.4 |
| Probing depths | -1.7 | 0.2 |
| Quality of life | -0.1 | 0.3 |
| Quantified levels of gingivitis | -0.5 | 0.5 |
| Quantified levels of plaque | -0.4 | 0.4 |
| Recession | -0.8 | -0.3 |
| Satisfaction with actual care received | 0.0 | 0.0 |
| Satisfaction with provider of care | -0.4 | -0.2 |
| Self-efficacy beliefs | -0.4 | -0.4 |
| Tooth loss | -0.5 | 0.6 |

Legend: A measure of the average change in score between each round of the e-Delphi study.
