# Supplementary material for: Developing a core outcome set for periodontal trials
Source: PLoS One. 2021 Jul 22;16(7):e0254123. doi: 10.1371/journal.pone.0254123 (PMC8297801; doi:10.1371/journal.pone.0254123)
Supplement: S7 Table — A measure of the change in an individual’s scoring between each round of the e-Delphi. (DOCX) [file pone.0254123.s008.docx]

**S7 Table. Stability of opinion scores between e-Delphi round 1 and 2 for each patient participant.**

| **Patient individual stability of opinion score** | |
| --- | --- |
| PERIO00022 | 0.4 |
| PERIO00028 | 0.5 |
| PERIO00031 | 1.6 |
| PERIO00033 | 0.7 |
| PERIO00037 | 0.2 |
| PERIO00044 | 0.0 |
| PERIO00046 | 0.2 |
| PERIO00049 | 0.1 |
| PERIO00052 | 0.6 |
| PERIO00059 | 0.2 |
| PERIO00061 | 0.2 |
| PERIO00066 | 0.6 |
| PERIO00072 | 1.1 |
| **Overall mean** | 0.5 |
| **Overall median** | 0.4 |

Legend: A measure of the change in an individual’s scoring between each round of the e-Delphi.
