# Supplementary material for: Developing a core outcome set for periodontal trials
Source: PLoS One. 2021 Jul 22;16(7):e0254123. doi: 10.1371/journal.pone.0254123 (PMC8297801; doi:10.1371/journal.pone.0254123)
Supplement: S8 Table — Voting scores for ‘top 10’ outcomes with number and percentage of stakeholder groups scores presented. Consensus for inclusion was pre-determined as 70% of participants. (DOCX) [file pone.0254123.s009.docx]

**S8 Table. Face to face consensus voting breakdown**

| Outcome | Total participants n=14 | | Patient participants n=8 | | Dental professionals n=6 | |
| --- | --- | --- | --- | --- | --- | --- |
|  | Yes (%) | No (%) | Yes (%) | No (%) | Yes (%) | No (%) |
| Bone levels on radiograph | 5 (36) | 9 (64) | 4 (50) | 4 (50) | 1 (17) | 5 (83) |
| Probing depths | 13 (93) | 1 (7) | 7 (87) | 1 (13) | 6 (100) | 0 (0) |
| Tooth loss | 12 (86) | 2 (14) | 6 (75) | 2 (25) | 6 (100) | 0 (0) |
| Compliance | 5 (36) | 9 (64) | 4 (50) | 4 (50) | 1 (17) | 5 (83) |
| Furcation involvement | 0 (0) | 14 (100) | 0 (0) | 8 (100) | 0 | 6 (100) |
| Quantified levels of plaque | 14 (100) | 0 (0) | 8 (100) | 0 (0) | 6 (100) | 0 (0) |
| Quantified levels of gingivitis | 14 (100) | 0 (0) | 8 (100) | 0 (0) | 6 (100) | 0 (0) |
| Smoking status | 2 (14) | 12 (86) | 2 (25) | 6 (75) | 0 (0) | 6 (100) |
| Quality of life | 12 (86) | 2 (14) | 8 (100) | 0 (0) | 4 (67) | 2 (33) |
| Clinical attachment loss | 3 (21) | 11 (79) | 1 (13) | 7 (87) | 2 (33) | 4 (67) |

Legend: voting scores for ‘top 10’ outcomes with number and percentage of stakeholder groups scores presented. Consensus for inclusion was pre-determined as 70% of participants.
