# Supplementary material for: Developing a core outcome set for periodontal trials
Source: PLoS One. 2021 Jul 22;16(7):e0254123. doi: 10.1371/journal.pone.0254123 (PMC8297801; doi:10.1371/journal.pone.0254123)
Supplement: Attachment — Submitted filename: Response to reviewers.docx [file pone.0254123.s011.docx]

| Dear Peter and reviewers,  Thanks for your feedback and suggestions to make our paper better for the reader. Please find a response to each point below. | |
| --- | --- |
| **Comments and suggestions** | **Response** |
| **Additional Editorial Comments** | |
| 1. Recently data have been published on the issue of end points in periodontal research that the authors at least may wich to discuss in their manuscript: e.g. Tomasi & Wennstrom 2017 and Feres et al. 2020. | Thank you for highlighting this work. We have added comment to the discussion section on line 308 *of ‘revised manuscript with tracked changes’.* |
| **Reviewer 1** | |
| This is an interesting study that has significance to the field of clinical studies in periodontology. The rationale for doing the study is reasonable. Methods used are appropriate for a study of this kind. Results are clearly presented. Conclusion are reasonable. | |
| 1. The sample size seems to be very small. I understand that sample size calculations were not possible but some comment on the sample size would be helpful. | As we stated in the manuscript there is no commonly accepted methodology for sample size calculations for e-Delphi studies.  Guidance on using Delphi methods in research recommends that “The Delphi group size does not depend on statistical power, but rather on group dynamics for arriving at consensus among experts. Thus, the literature recommends 10–18 experts on a Delphi panel”.   - *Chitu Okoli, Suzanne D. Pawlowski, The Delphi method as a research tool: an example, design considerations and applications, Information & Management, Volume 42, Issue 1, 2004, Pages 15-29,* - *Paliwoda, S.J. (1983), "Predicting the Future Using Delphi", Management Decision, Vol. 21 No. 1, pp. 31-38. https://doi.org/10.1108/eb001309*   We understand that this could be a common question and have added further clarification to line 138 of ‘*revised manuscript with tracked changes’.*  The aim of the e-Delphi study in the context of Core Outcome Sets (COSs) is to prioritise outcomes to be taken forward to the face-to-face consensus meeting. We therefore took a pragmatic approach to selecting sample size which is similar to other COSs using e-Delphi methodology.   - *KARAS, J., ASHKENAZI, S., GUARINO, A., LO VECCHIO, A., SHAMIR, R., VANDENPLAS, Y. & SZAJEWSKA, H. 2015. A core outcome set for clinical trials in acute diarrhoea. Archives of Disease in Childhood, 100, 359-363.* - *SINHA, I. P., GALLAGHER, R., WILLIAMSON, P. R. & SMYTH, R. L. 2012. Development of a core outcome set for clinical trials in childhood asthma: a survey of clinicians, parents, and young people. Trials, 13, 103.*   We have added comment to line 348 (of *revised manuscript with tracked changes*) to provide further clarity to the reader. |
| 2. In line with the above comment a response rate of 61 dental professionals with a final participant rate of 51 dental professionals (across all facets of dentistry) seems very low and deserves some comment. Likewise what I s the likelihood of bias in the responses of the motivated dental professionals who participated of this study? | As outlined above there is no commonly accepted methodology for sample size. We feel that our sample involves representation from across the dental profession. A number of our professional participants fulfil a number of roles and we record their primary roles in table 2. We have highlighted the limitations of our overall participant cohort in our discussion. A large number of the dental professional participants were recruited via the British Society of Periodontology, some of whom will have been involved in international meetings and consensus exercises. |
| 3. I am surprised that the people in the Patient Group could understand most if not all of the “clinical” outcomes. It is not clear how this was controlled for in the study. For example how many of these people really understood the meaning or significance of “furcation involvement”, functional occlusion”, "clinical attachment loss” etc etc? | Context and further information added to results (line 268) and limitations section of discussion (line 362). However, the face to face meeting allowed full discussion of outcomes and greater depth of understanding – comment added to strengths of study (line 285 & 339).  All lines quoted are from *‘revised manuscript with tracked changes’* |
| **Reviewer 2** | |
| Overall, this work addresses a major issue in current periodontal studies: standardized reporting of the disease to make research work comparable. This study has been registered at COMET (http://www.comet-initiative.org/studies/details/265?result=true) and a detailed and understandable method paper has been published elsewhere: doi: 10.1186/s13063-017-2169-z. However, there are some major issues, that need consideration from the author. | |
| Major revisions | |
| 1. Page 3, line 43: It is hard to understand, why Quality of Life is the core outcome, instead of oral health related Quality of Life. The selection of the variables, as well as the final decision is not comprehensible for the reader. | It is difficult to gain that depth of understanding for the reader in the abstract alone.  The COS development process brings the stakeholders together to reach consensus on what outcomes should be measured. As outlined in the conclusion section the next phase would be ‘how’ these outcomes should be measured. Quality of life may well be measured as oral health related quality of life in the future, but that would be considered the next phase of work – following the “Consensus-based standards for the selection of health Measurement Instruments (COSMIN) initiative” methodology.  For clarity both quality of life and oral health related quality of life have previously been reported in the literature. We understand the instruments used can measure very different things but we felt this would be decided by consensus using the COSMIN methodology. To address this point and comment 15 below we have now added full dataset to the appendix to ensure that this is now fully transparent (*S2 Table to S5 Table*). |
| 2. Page 4, line 46-47: “Implementation of this COS will ensure the results of effectiveness trials for periodontal diseases are more relevant to patients and dental professionals. , reducing research wastage.“ In general, the author should reconsider the wording: research wastage, because it implies that all research done so far is wastage. | It is not our intention to imply that all previous research is wasteful. We do not think that stating that the that COS will ensure results are more relevant does imply this. We have added “future” to the sentence to hopefully clarify this:  *“Implementation of this COS will ensure the results of* ***future*** *effectiveness trials for periodontal diseases are more relevant to patients and dental professionals, reducing research wastage.“* |
| 3. Page 4, line 46-47: The author should consider to explain how the research outcome leads to “…uncertainty for patients…”. | Text added to line 51 (of *‘revised manuscript with tracked changes’)*: “This could reduce uncertainty for patients and dental professionals by ensuring the evidence used to inform their choices is meaningful to them. It could also strengthen the quality and certainty of the evidence about the relative effectiveness of interventions.” |
| 4. Page 5, line 64: Is there a difference in “Quantified levels of gingivitis; Quantified levels of plaque“ and “Gingivitis; Plaque“? | No, there is no difference. The language used was taken from the outcomes identified in the literature review. |
| 5. The introduction is well written and the author provides the reader with all the relevant information to understand the objective of the study. | Thank you. |
| 6. Page 6, line 80: The author cites six papers to prove outcome heterogeneity in periodontal trials. However, all six papers investigate oral health care approaches for periodontal health. None of the papers are actually performing or reviewing periodontal treatment and its outcome. Please reconsider citation. Also consider some epidemiological approaches: doi: 10.1111/jcpe.12392. | Thank you for your suggested citations. We have updated the citations to include the suggested papers:   - *Feres M, et al. Proposal of a Clinical Endpoint for Periodontal Trials: The Treat-to-Target Approach. J Int Acad Periodontol. 2020 Apr 1;22(2):41-53. PMID: 32224549*. - *Holtfreter B, Albandar JM, Dietrich T, Dye BA, Eaton KA, Eke PI, Papapanou PN, Kocher T; Joint EU/USA Periodontal Epidemiology Working Group. Standards for reporting chronic periodontitis prevalence and severity in epidemiologic studies: Proposed standards from the Joint EU/USA Periodontal Epidemiology Working Group. J Clin Periodontol. 2015 May;42(5):407-12. doi:* *10.1111/jcpe.12392. Epub 2015 May 7. PMID: 25808877; PMCID: PMC7441325*.   The original citations include a clinical guidance document covering prevention and treatment of periodontal disease. The guidance developers included systematic reviews of the literature, and appraised the literature in their clinical recommendations. We think these citations are all appropriate to include – a number of Cochrane systematic reviews and Clinical Guidelines highlight the issue of outcome heterogeneity. |
| 7. Page 6, line 83: Please provide citation. | Citations added:   - *Alshamsi, M., J. Mehta, and L. Nibali, Study design and primary outcome in randomized controlled trials in periodontology. A systematic review. Journal of Clinical Periodontology, 2021. 48(6): p. 859-866.* - *Glasziou, P., et al., Reducing waste from incomplete or unusable reports of biomedical research. Lancet, 2014. 383(9913): p. 267-76.* |
| 8. Page 6, line 84-85: Why does the author only refer to interventional trials? The title addresses all periodontal trials? Please specify, whether the current work provides outcome for interventional and observational studies. The current literature does provide some agreeable outcomes for periodontal diseases, e.g. for clinical or epidemiological approaches: doi: 10.1002/JPER.18-0006 and doi: 10.1902/jop.2016.160379. | Line 92-95 (of *‘revised manuscript with tracked changes’)* explains that The COMET (Core Outcome Measures in Effectiveness Trials) initiative develops core outcome sets (COS), that are defined as an agreed, standardised collection of outcomes which should be measured and reported in all **effectiveness** trials for a specific clinical area or intervention. By definition the COS are developed for interventional/effectiveness trials.  We attempted to keep the title as succinct as possible. We could consider changing to “developing a core outcome set for effectiveness trials investigating the prevention and management of periodontal diseases” if the editorial team think that would address these issues? |
| 9. Page 7, line 98: “This COS would not be limited by health status, age or clinical setting.” Is this a second hypothesis? Please rephrase or relocate to Results. | To confirm this is not a second hypothesis. We are setting out the scope of the COS – which is recognised as good practice to do for COS development:  *Williamson, P.R., Altman, D.G., Bagley, H. et al. The COMET Handbook: version 1.0. Trials 18, 280 (2017). https://doi.org/10.1186/s13063-017-1978-4.* |
| 10. Page 7, line 99-100: “Our report is in accordance with recommendations of the Core Outcome Set-STAndards for 100 Reporting (COS-STAR) checklist.“ Please relocate to M&M. | Moved to M&M. |
| 11. Page 8, line 117: The author included 11 studies (review/protocol) from the Cochrane database. Why did the author limit the search to the Cochrane database? From the selected studies, only 1 study investigates outcomes after periodontal treatment. Why did the author decide to include 10 studies comparing oral health interventions and only one focusing on periodontal treatment? Inclusion criteria for literature search is not properly stated. | The COMET handbook (reference comment 9 above) advises that *“The scope of the review should be carefully considered in the context of the COS to ensure that outcomes are included from all relevant studies* ***without unnecessary data collection****.”*  The ‘identifying existing knowledge stage’ should involve a pragmatic but admittedly not exhaustive search of the existing outcomes previously reported. This search should identify the key outcomes previously reported but by its nature will likely only include outcomes important to researchers/clinicians. This potential shortcoming is addressed by asking participants of the Delphi study to suggest missing outcomes – either missing from our search (i.e. reported in other published studies) or outcomes that have not previously been investigated. We had 68 individual suggestions, which when de-duplicated resulted in 34 unique suggestions.  We focused our search on Cochrane reviews as a pragmatic way to identify outcomes previously reported in effectiveness trials. We included all relevant Cochrane reviews on the Cochrane database at the time of the search (July 2016). There two dental professional delivered intervention reviews “Routine scale and polish for periodontal health in adults” and “Full-mouth treatment modalities (within 24 hours) for chronic periodontitis in adults” and these interventions are traditionally delivered in combination with the ‘self-care’ interventions, the reviews of which were also included. The results of our review identified that previous trials focused on surrogate outcome measures of periodontal diseases. This was a pragmatic approach that could be added to during the next stage “filling in gaps in knowledge”. |
| 12. Page 9, line 132: What are the exact numbers for dental professionals (n=?) and researchers (n=?)/ or refer to Table 2. | Page 9, line 132 is the methods section so we do not present the results at this point. We have highlighted the breakdown of participant numbers in the relevant part of the results section: page 16, line 251-255 and Table 2.  We did not specifically set individual targets for recruitment of dental professionals vs researchers. A large number of professionals would fill multiple roles (dentists, specialists, academic, statistician). Our target sample size was therefore based on ensuring adequate representation of patients and across professional groups. Please also see comments above to comments 1 & 2 of reviewer one. |
| 13. Page 9, line 136: The author states that there were no absolute exclusion criteria. But what about language barriers? | We did not include ability to speak English as an exclusion criteria. |
| 14. Page 9, line 139: Maybe the author could provide this as supplementary material? | That is a good idea thanks. We have uploaded as Supplementary material – S1 Appendix. |
| 15. Page 14, line 226: The author is providing supplementary Material: Appendix A. The table lists the literature the author chose to provide sufficient outcome variables. Please check citation – where can the reader find the full citation? | We have added the full dataset to supporting information - *S2 Table to S5 Table*. |
| 16. In the Methods Paper (doi: 10.1186/s13063-017-2169-z) the author states that the literature research has been conducted up to July 2016 – here the author states “The search was conducted in July 2016.” – please clarify. | It was up to July 2016. We have updated the language accordingly. |
| 17. I understand the struggle of conducting such a complex mixed method study involving literature review; online Delphi Study; and face-to-face consensus meeting. However, now in 2021 the literature included is at least 5 years old. I would suggest to address this as a limitation. | Whilst we understand the question, we do not think that there has been a change in outcomes reported or interventions tested over this time period. This search was conducted to identify previously reported outcomes – the clinical outcomes reported in the periodontal research have been remarkably stable for decades. They have been identified in our search and included in our COS development. We therefore do not see this as a limitation of the study. |
| 18. Page 15, line 260: The step from round 2 to round 3 is a bit difficult to understand. For example: clinical attachment loss (CAL) were included from both dental professionals/researchers and the patient group with 90% and 89%, respectively. But in round 3, in the final discussion, CAL were excluded as final outcome. Why? CAL is a much more reliable measurement for periodontitis instead of probing depth, because it considers gum recession. Can the author provide any plausible numbers in table 3 for round 3? | We note ‘clinical attachment loss’ use as an outcome for both clinical practice and research. It was rated highly during the Delphi study by both patient and professionals but during the face-to-face meeting with professionals and patient the group did not elect to include this in a **core** outcome set.  It is hard in a manuscript like this to discuss the depth of discussion for every single outcome considered. We have added further detail to line 293 (of *‘revised manuscript with tracked changes’)* and a further table added supporting information - *S9 Table* presenting the voting scores for the final 10 outcomes considered in this process. |
| 19. Page 16, line 269: Quantified levels of gingivitis is one of the five core outcomes. How was gingivitis classified? The aim of the current work is to provide standardized outcomes in order to prevent “research wastage” – then the core outcomes must be clearly and understandable described in order for reproduction by other authors. | The aim of COS development is gaining consensus on what outcomes should be investigated “the what”. The next stage is “the how”. As outlined line 396 (of *‘revised manuscript with tracked changes’)* this should be determined by following the methodology suggested by ‘Consensus-based standards for the selection of health Measurement Instruments (COSMIN) initiative’. |
| 20. In the first paragraph of the discussion the author should summarize the main findings of the study. | We have edited the initial text of the discussion. We are not sure about the added value of repeating the final COS in this paragraph as it has been presented in the previous paragraph (final paragraph of the results section). |
| 21. Page 16, line 275: “There are however a small number developed or in progress across dentistry [26-28]. “Did they reported similar outcomes? Please compare main results of your study. | The other COSs have been developed for other specialties of dentistry. There is heterogeneity of remit and scope. These COS developers have reported similar methodologies but we are not sure of the value of comparing our outcomes/results to theirs. In some ways it would be like comparing our results to COS for diabetes. Although they are dental COS it is different diseases/scope being considered. |
| 22. Page 17, line 289-291: What does the author mean with “The scope of the study (both interventions and health area)…“? Also “…outcomes for all trials that investigate the prevention and management of periodontal diseases in effectiveness trials“. In conclusion, the author recommends the outcomes only for interventional studies? This part is unclear in the introduction and in the title. | Please see response to comment 8. We feel this issue has been explained in the introduction and would be open to title change. |
| 23. The author states in the Introduction that “This COS would not be limited by health status, age or clinical setting.“. However this statement is not taken up again in the results section or in the discussion. Please clarify. | Please see reply to comment 9 above. We have also added text confirming this to line 331 of *‘revised manuscript with tracked changes’.* |
| Minor revisions | |
| 1. Page 6, line 77-78: Please provide citation. | Citations added:   - *West, N., et al., BSP implementation of European S3 - level evidence-based treatment guidelines for stage I-III periodontitis in UK clinical practice. Journal of Dentistry, 2021. 106: p. 103562.* - *Sanz, M., et al., Treatment of stage I–III periodontitis—The EFP S3 level clinical practice guideline. Journal of Clinical Periodontology, 2020. 47(S22): p. 4-60.* - *(SDCEP), S.D.C.E.P., Prevention and Treatment of Periodontal Diseases in Primary Care. 2014, Dundee: Scottish Dental Clinical Effectiveness Programme.* - *Delivering better oral health: an evidence-based toolkit for prevention* |
| 1. Page 6, line 78: “There is a wide variety…” instead of “There are a wide variety…”. | Updated. |
| 2. Page 16, line 270: “… Quantified levels of plaque, Tooth loss (Table 4). “ | Updated. |
